# Polymorphisms in Fatty Acid Desaturase 2 Gene Are Associated with Milk Production Traits in Chinese Holstein Cows

**DOI:** 10.3390/ani10040671

**Published:** 2020-04-12

**Authors:** Mingxun Li, Qisong Gao, Mengqi Wang, Yan Liang, Yujia Sun, Zhi Chen, Huimin Zhang, Niel A. Karrow, Zhangping Yang, Yongjiang Mao

**Affiliations:** 1 Key Laboratory of Animal Genetics & Breeding and Molecular Design of Jiangsu Province, Yangzhou University, Yangzhou 225009, China; limingxun@live.com (M.L.); MZ120181011@yzu.edu.cn (Q.G.); mengqi.wang.1@ulaval.ca (M.W.); 15755081060@163.com (Y.L.); chenzhijerom@163.com (Z.C.); minmin-911@163.com (H.Z.); yzp@yzu.edu.cn (Z.Y.); 2 Joint International Research Laboratory of Agriculture and Agri-Product Safety of Ministry of Education of China, Yangzhou University, Yangzhou 225009, China; ysunshine30@outlook.com; 3 Center for Genetic Improvement of Livestock, Department of Animal Biosciences, University of Guelph, Guelph, ON N1G 2W1, Canada; nkarrow@uoguelph.ca

**Keywords:** fatty acid desaturase 2, single nucleotide polymorphisms, milk production traits, somatic cell score

## Abstract

**Simple Summary:**

Searching for causative polymorphisms underlying the variability of milk production traits and then incorporating them into breeding programs are very effective ways to improve the efficiency and reliability of conventional dairy cattle breeding. Fatty acid desaturase 2 (FADS2) plays a pivotal role in the biosynthesis of polyunsaturated fatty acids. Previous studies provided evidence that *FADS2* was one of the most downregulated genes during negative energy balance in the liver of postpartum dairy cattle. Genes involved in the energetic pathways may influence other production traits, such as protein, fat and milk yields. Therefore, in the present study, we investigated the common genetic variants of the *FADS2* gene in Chinese Holstein cows. Our results provided direct evidence that *FADS2* was an interesting candidate for selection to increase milk production and improve resistance against mastitis.

**Abstract:**

This study investigated the single nucleotide polymorphisms (SNPs) of Fatty acid desaturase 2 (*FADS2*) gene and further explored their genetic effects on conventionally collected milk production traits in Chinese Holstein cows using 18,264 test-day records of 841 cows. One missense mutation c. 908 C > T (SNP site in the complementary DNA sequence), which caused an amino acid change from alanine to valine (294Ala > Val), and two 3’ untranslated region (UTR) SNPs, c.1571 G > A and c.2776 A > G were finally identified. The SNP c.908 C > T was significantly associated with test-day milk yield, fat percentage and 305-day milk, fat and protein yield. In particular, the T allele of the SNP c.908 C > T showed a significant association with decreased somatic cell score (SCS) in the investigated population. Significant relationship between the SNP c.1571 G > A and 305-day milk yield showed that genotype GG was linked to the highest milk yield. Substituting the allele G for A at the c.2776 A > G locus resulted in a decrease of protein percentage. Our results demonstrated that *FADS2* was an interesting candidate for selection to increase milk production and improve resistance against mastitis.

## 1. Introduction

Advances in the determination of genetic variants and chromosomal regions influencing economically important traits provide new opportunities for the improvement of milk production traits in dairy cattle [1]. Searching for causative polymorphisms underlying the variability of milk production traits and then incorporating them into breeding programs are very effective ways to increase the efficiency and reliability of conventional dairy cattle breeding [2]. Genome-wide association studies (GWAS) and candidate gene approach are two main strategies for studying the genetic architecture of complex traits [3]. Both approaches have their advantages and limitations. Broadly, genome-wide association studies involve scanning common variation encompassing the entire genome, and as such can pinpoint genes regardless of whether their functions were known [4], but it is expensive and resource intensive, while the candidate gene approach is more powerful and more straightforward for the genetic dissection of complex traits, but it is limited by its reliance on existing knowledge about the molecular mechanisms that contribute to phenotype [5]. At present, many candidate genes involved in the development of dairy cow mammary gland and lactation processes have been identified as affecting milk production and composition, such as diacylglycerol acyltransferase 1 (*DGAT1*), stearoyl-CoA desaturase (*SCD*), fatty acid-binding protein-4 (*FABP4*) and fatty acid desaturase 2 (*FADS2*) [6,7,8,9].

The *FADS2* gene encodes a crucial enzyme of long-chain polyunsaturated fatty acids (LC-PUFAs) biosynthesis able to catalyze the introduction of double bonds at the sixth carbon atom in a large spectrum of fatty acids [10,11]. Loss of FADS2 expression in the FADS2-deficient mouse impeded the processing of essential fatty acids linoleic (C18:2n-6) and α-linolenic acid (C18:3n-3) to n-6/n-3 LC-PUFAs, demonstrating that FADS2 is the only enzyme that catalyzes this pivotal step [12]. The FADS2^-/-^ liver exhibited severe changes in the phospholipid-bilayer structures of subcellular membranes, which disturbed the maturation of transcription factor sterol regulatory element-binding protein (SREBP1c), and therefore perturbed lipid metabolism [13].

The bovine *FADS2* gene has been proposed as a candidate gene influencing milk fatty acids composition [9,14,15], a primary aspect of milk nutritional quality [16]. Bovine *FADS2* is comprised of 12 exons encoding 359 amino acid chains and is located on bovine chromosome 29 (BTA29) in the region 29q17-29q18, a region associated with fatty acid content and respiratory diseases susceptibility. In addition to *FADS2*, *FADS1* and *FADS3* are also clustered at the same genomic locus. Multiple single nucleotide polymorphisms (SNPs) have been determined in the bovine *FADS*-gene cluster. Ibeagha-Awemu et al. described the genetic diversity within *FADS1* and *FADS2* genes, and demonstrated significant associations between three SNPs with two milk n-6 LC-PUFAs, dihomo-gamma-linolenic acid (DGLA, C20:3n-6) and arachidonic acid (ARA, C20:4n-6), and one n-3 LC-PUFA, eicosapentaenoic acid (EPA, C20:5n-3) of Canadian Holstein cows [9]. Takahashi et al. reported that the SNP rs211580559 in exon 7 of the *FADS2* gene had a significant effect on intramuscular linoleic acid (LA, C18:2n-6) composition in Japanese Black Steers [17]. In a transcriptomic study, Wang et al. produced evidence that *FADS2* was a strong candidate gene related to intramuscular fat deposition [18]. Recently, Proskura et al. revealed significant relationships between the SNP rs209202414 in intron 3 of the *FADS2* gene and milk eicosatrienoic acid (ETA, C20:3n-3) and docosadienoic acid (DDA, C22:2n-6) in Jersey cows [19].

The precedent results revealed the associations of the *FADS2* SNPs with milk fat traits in dairy cattle. However, much remains unknown about the effects of the *FADS2* polymorphisms on routinely collected milk production traits [9,19]. Previous studies provided evidence that *FADS2* was one of the most downregulated genes during negative energy balance in the liver of postpartum dairy cattle [20,21]. It is reasonable to assume that genes involved in the energetic pathways may influence other production traits, such as protein, fat and milk yields, which represent the principal breeding objectives of the current selection programs. Therefore, in the present study, we investigated the common genetic variants of the *FADS2* gene in Chinese Holstein cows, explored the effects of genetic polymorphisms on milk production traits and further evaluated the average effects of allele substitution.

## 2. Materials and Methods

### 2.1. Animals and Milk Production Records

From June 2010 to December 2014, a total of 20,556 milk samples from 2558 lactations (lactations 1 to 5) of 841 cows reared in Yancheng city of China, were collected during monthly test-day milk recording. These cows were daughters of 162 sires, with 2 to 43 daughters per sire, housed in free stalls, milked three times per day and fed a total mixed ration (TMR). Milk samples were analyzed in the Dairy Herd Improvement laboratory of Shanghai Dairy Cattle Breeding Center using Milko-Scan FT6000 (Foss Electric, Denmark). Values of somatic cell count (SCC) were determined with a Fossmatic 5000 cell counter (Foss Electric, Denmark). Somatic cell score (SCS) were calculated using the formula: SCS = log2(SCC / 100,000) + 3 [22]. Only data with SCC between 1×10^3^ and 5×10^5^ were kept for further analyses. Following this criterion, a total of 18,264 test-day records were contained in this study. 

### 2.2. DNA Extraction and SNP Genotyping

Blood samples were collected from above mentioned 841 Chinese Holstein cows. Genomic DNA was extracted from the white blood cells using a standard phenol-chloroform procedure with a slight modification in centrifugation speed and time [23,24]. The DNA concentration was measured by NanoDrop 1000 spectrophotometer (Thermo Scientific, Wilmington, DE, USA) and evaluated for integrity by 1% agarose gel electrophoresis.

According to previous studies [9], three primer pairs were designed to screen genetic polymorphisms situated in the exon 7 and 3’ untranslated region (UTR) of *FADS2* in Chinese Holstein cows (Table S1). DNA samples from 20 cows were utilized for PCR amplification and sequencing to identify SNPs. The PCR reactions were carried out in a PTC-200 DNA Engine cycler (Bio-Rad, CA, USA) using an optimal annealing temperature (Table S1) determined by a PCR temperature gradient. Twenty microliters of PCR amplicons were sequenced in Shanghai Sangon Company (Shanghai, China) using an ABI PRISM 3700 DNA Sequencer (Applied Biosystems, Foster City, CA, USA). After sequencing, the forward and reverse sequences were assembled using the ContigExpress module in Vector NTI Advance 11.5 (Invitrogen, Carlsbad, CA, USA) to discover novel SNPs. Animal genotyping for the discovered SNPs was carried out with the MassARRAY system (Sequenom Inc., San Diego, CA, USA) [25], which uses a matrix-assisted laser desorption ionization-time of flight mass spectrometry platform (MALDI-TOF). 

### 2.3. Statistical Analyses

The allele and genotype frequencies were directly calculated. The linkage disequilibrium (*r^2^*) were measured for all SNPs pairs using the SHEsis software [26]. Association studies were carried out using the following linear model:

y*_ijklmn =_* μ + Sire*_i_* +T*_j_*+ parity*_k_* + DIM*_l_* + G*_m_* + e*_ijklmn_* (1)

where y is the phenotypic value for the analyzed trait; μ is the overall mean; Sire*_i_* is the fixed effect of the *i*th sire; T*_j_* is the fixed effect of tested year and season of calving; parity*_k_* is the fixed effect of the parity (three classes: parity 1, 2 and 3–5); DIM*_l_* is the fixed effect of the stage of lactation (10 levels of 30 d each); G*_m_* is the fixed effect of the *m*th genotype; and e*_ijklmn_* is the random residual.

The allelic additive (*a*), dominance (*d*) and substitution (*α*) effects were estimated using the equation: *a* = (AA − BB)/2, *d* = AB − (AA + BB)/2 and *α* = *a* + *d* (*q* − *p*), where AA and BB represent the phenotypic value of two homozygous genotypes, AB represents the phenotypic value of heterozygous genotype and *p* and *q* are the corresponding allele frequencies of A and B [27]. 

## 3. Results

### 3.1. SNP Detection

**Figure 1:**
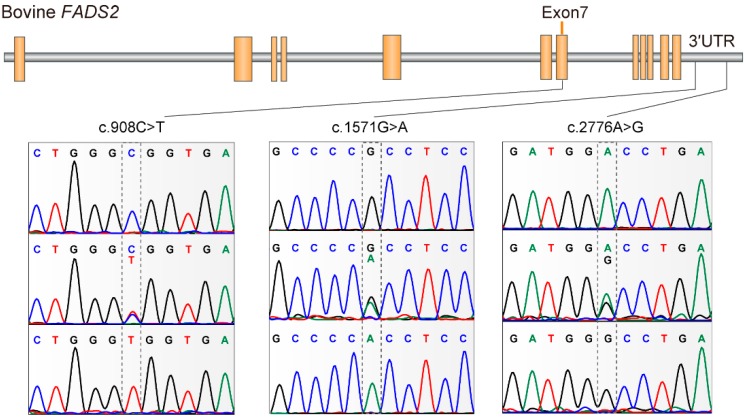
Schematic diagram of the *fatty acid desaturase 2* gene with the localization of the three identified single nucleotide polymorphisms (SNPs).

Investigation of the exon 7 and 3’ UTR sequences of *FADS2* evidenced the presence of three SNPs, including one SNP c.908 C > T (rs211580559) located within the protein coding sequence, and two SNPs in the 3’ UTR, c.1571 G > A (rs210169303) and c.2776 A > G (rs207932003). Details of the three segregating SNPs including the location on BTA29 relative to *FADS2* were illustrated in Figure 1. The SNP c.908 C > T was a missense mutation that caused an amino acid change from alanine to valine (p. Ala294Val). TargetScan analysis suggested that the SNP c.1571 G > A was located within the binding site for bta-miR-744. The presence of the minor allele A abolished the ability of miR-744 to bind *FADS2*.

### 3.2. Genetic Diversity Analyses

The allele and genotype frequencies are presented in Table 1. The minor allele frequency (MAF) for the three segregating SNPs ranged from 0.107 (c.1571 G > A) to 0.407 (c.908 C > T). Linkage disequilibrium was measured by *r^2^* between all the SNP pairs. The *r^2^* values between c.908 C>T and c.1571 G > A, c.908 C > T and c.2776 A > G and c.1571 G > A and c.2776 A > G were 0.054, 0.152 and 0.024, respectively. No significant evidence of linkage disequilibrium was detected between the loci in the investigated population. 

### 3.3. Associations of SNPs with Milking Traits and Somatic Cell Score

**Figure 2:**
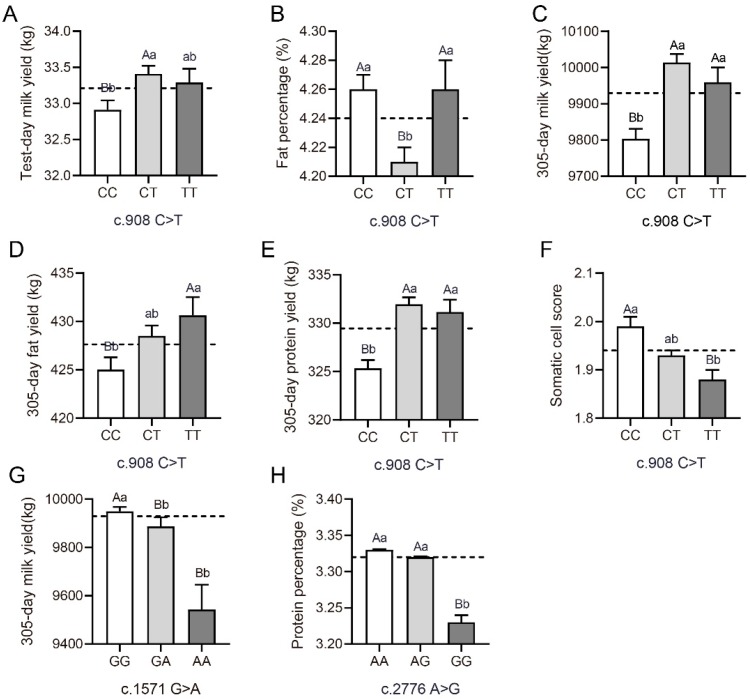
The effects of investigated *fatty acid desaturase 2* SNPs on milk production traits. (**A–F**) The SNP c.908 C > T was significantly associated with test-day milk yield, fat percentage and 305-day milk, fat and protein yield, and somatic cell score (*n =* 6553 for CC; *n =* 8687 for CT; and *n =* 3024 for TT). (**G**) The GG genotype at the c.1571 G > A locus was linked to the highest 305-day milk yield (*n =* 14578 for GG; *n =* 3313 for GA; and *n =* 373 for AA). (**H**) Substituting allele G for A at the c.2776 A > G locus resulted in a decrease of protein percentage (*n =* 12110 for AA; *n =* 5619 for AG; and *n =* 518 for GG). Different lowercase letters indicate significant differences between genotypes (*p* < 0.05); Different uppercase letters indicate significant differences between genotypes (*p* < 0.01). Data shown are means ± SE. The dotted line represents the overall mean.

**Figure 3:**
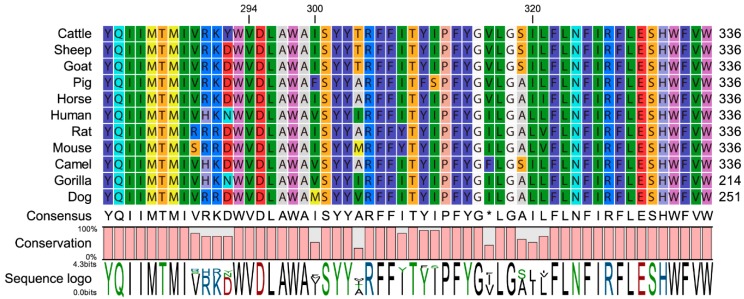
Multiple alignments of fatty acid desaturase 2 protein sequences from mammalian species. The position of Ala294Val mutation is indicated by the vertical line. The numbers on the right are relative positions of amino acid.

The effects of the investigated *FADS2* SNPs on milk production traits are presented in Figure 2 and Table S2. The SNP c.908 C > T, a missense mutation within the *FADS2* gene, was significantly associated with test-day milk yield, fat percentage and 305-day milk, fat and protein yield (Figure 2A–F). In particular, the TT cows yielded more milk, fat and protein for the entire 305-d lactation than CC animals. The milk from the CC cows was significantly richer in test-day fat (+1.19%) compared with CT genotype (4.26 vs. 4.21, Table S2). In the case of test-day milk yield, 305-day milk yield, and 305-day protein yield, the SNP c.908 C > T exhibited overdominance with the heterozygous genotype was greater than the two homozygous, confirmed by a |*d*/*a*| ratio higher than the threshold value of 1.2 [28]. This could be a result of heterozygotes experiencing advantageous effects, which is consistent with the highest genotype frequency exhibited by CT heterozygotes. A significant association was also observed between the c.908 C > T and SCS. In this case, the genotype TT was associated with the lowest SCS (Figure 2F). This result is supported by allele substitution analysis whereby substituting the allele T for C linked to a decrease of 0.05 of milk SCS to the allele T (Table S2). Protein sequence alignment of FADS2 showed that Val 294 was highly conserved among mammalian species including cattle, sheep, goat, pig, horse, human, rat, mouse, gorilla and dog (Figure 3).

Significant relationship between the SNP c.1571 G > A and 305-day milk yield showed that genotype GG was linked to the highest milk yield, although this significance disappeared after Bonferroni correction. Allele substitution at this locus (allele A substituted for G) indicated that allele A decreased milk yield by 313.76 kg (Figure 2G, Table S2). Substituting allele G for A at the c.2776 A > G locus resulted in a decrease of protein percentage (5%, *p* < 0.001, Figure 2H). In spite of this, no statistically significant differences were found in the 305-day protein yield at this locus.

## 4. Discussion

FADS2 plays a pivotal role in the biosynthesis of polyunsaturated fatty acids [29,30,31,32]. Genome-wide association studies (GWAS) have confirmed the effects of *FADS2* genetic variations on diseases related to lipid metabolism [33,34]. In domestic animals, Zhu et al. reported that single nucleotide polymorphisms (SNPs) in *FADS2* affected essential fatty acid content in muscle and the growth rate of early developing chickens [35]. Matsumoto et al. revealed that the *FADS2* g.-823G > A had significant effects on several beef quality traits including beef marbling score [36]. Boschetti et al. demonstrated that *FADS1/FADS2* genotypes are related to desaturating ability, with a significant impact on the PUFA content of chicken breast meat [37]. These findings confirmed that the *FADS2* gene is a strong candidate gene affecting the fatty acid composition.

In the present study, we investigated the genetic diversity of three identified SNPs in the *FADS2* gene and further explored their genetic effects on conventionally collected milk production traits in Chinese Holstein cows. Genotype distribution demonstrated that the allele frequencies at the c.908 C > T locus were in agreement with previous findings carried out in Canadian Holstein cows [9], whereas the G allele frequency at the c.1571G > A locus was slightly higher in our investigated Chinese Holstein population (0.893 vs. 0.731) [9]. This might be attributed to several reasons including different breeding objectives that favored the G allele in Chinese Holstein cows, and smaller population size or simply the greater number of farms involved in the previous study.

Significant associations were recorded between *FADS2* polymorphisms and milk production traits. The SNP c.908 C > T was associated with higher milk, fat and protein yields, confirming an important role of the *FADS2* gene in affecting milk production traits. Moreover, the T allele of the SNP c.908 C > T showed a significant association with decreased somatic cell score (SCS) in the investigated population. Currently, the dairy cows have been subjected to intense selection for milk yield which has adversely affected cow health [38,39]. The decreased genetic trend in cow health is becoming a major concern and the focus of selective breeding has shifted from a production-oriented to a more balanced breeding goal. Traits encompassing health, such as mastitis resistance, have now been incorporated into selection programs. Genetic evaluation and selection for decreased SCS can decrease mastitis incidence in dairy cattle populations [39]. In previous studies, Vesna et al. have described that α-linoleic acid (ALA) supplementation decreased somatic cell count of dairy goats [40]. Greco et al. reported that altering the dietary n-6 to n-3 ratio influenced cow lactation performance and SCC to an LPS challenge [41]. Given these findings and the anti-inflammatory effects of n-3 fatty acids [42], it is reasonable to assume that the genes associated with n3/n6 PUFA profiles may have potential effects on immune responses and somatic cell count. 

The SNP c.908 C > T was a missense mutation that caused an amino acid change from alanine to valine (294Ala > Val). Multiple alignments of *FADS2* protein sequences demonstrated that Val 294 was highly conserved among mammalian species, implying its importance for *FADS2* protein function. Takahashi et al. reported that the SNP c.908 C > T had a significant effect on intramuscular linoleic acid content in Japanese Black Steers [17]. However, in another study, this SNP has not exhibited a functional consequence, with no significant effects on any of the investigated milk fatty acid profiles in dairy cattle [9]. The Val and Ala are both hydrophobic amino acids with aliphatic side chain groups. This replacement in amino acids does not appear to cause the structural and functional change of *FADS2* protein. Therefore, the biological mechanism of how this missense mutation affected milk production traits requires to be further elucidated.

The SNP c.1571 G > A resulted in a decrease of 305-day milk yield. In Canadian Holstein cows, this SNP has been associated with milk n-6 fatty acids, C18:2n10t12c and C18:2n6tt, before FDR correction [9]. However, in the present study, no significant association was found between the SNP c.1571 G > A and any of fat related traits, such as test-day fat content and 305-day fat yield. The SNP c.1571 G > A is located in the 3’ UTR of *FADS2* gene. There is conclusive evidence that 3’ UTR sequences participated in gene expression regulation through different molecular mechanisms, including miRNA binding, polyadenylation and RNA stability [43]. Bioinformatics analysis suggested that the presence of the minor allele A abolished the ability of miR-744 to bind *FADS2*. These findings provide additional support to the proposition of *FADS2* as a candidate gene for further research to unravel the mechanisms by which it influences milk production traits.

RNA-seq analysis showed that *FADS2* was the most significantly downregulated gene in the liver of severe negative energy balance cows, indicating that *FADS2* is a potential gene that may play a crucial role in metabolic adaptations to negative energy balance in high-yielding dairy cows [21]. Given that the milk synthesis is energetically costly [44,45], it is reasonable to hypothesize that genes involved in the energetic pathways can also affect milk production traits, such as protein, fat and milk yields. This relationship is partially confirmed by previous findings that the alanine variant of the DGAT1 p.Lys232Ala polymorphism and the tyrosine variant of the GHR p.Phe279Tyr polymorphism that were reported to have favorable effects on effective energy balance accumulating throughout the lactation period are associated with increased milk production [46,47,48]. These findings may explain why *FADS2* polymorphisms were significantly associated with milk production traits.

## 5. Conclusions

To the best of our knowledge, this is the first study to estimate the effects of *FADS2* polymorphisms on milk production traits in Chinese Holstein cows. Our results provided direct evidence that *FADS2* was an interesting candidate for selection to increase milk production and improve resistance against mastitis. Specifically, the TT genotype at the c.908C > T locus was the most desirable to select for animals producing higher quality and healthier milk because that TT genotype was significantly associated with higher 305-day milk, fat and protein yield and a lower SCS. Further experimentations are required to validate the role of identified SNPs on milk production traits in other populations and breeds before applying them in gene-assisted selection in Holstein cow.

## Figures and Tables

**Table 1:** The allele and genotype frequencies, and Hardy–Weinberg equilibrium test for the SNPs in the *fatty acid desaturase 2* gene in Chinese Holstein cows.

Locus	Allele	Allele Frequency	Genotype	Genotype Frequency	Observed Count	Expected Count
c.908 C > T	C	0.593	CC	0.351	295	295.48
	T	0.407	CT	0.484	407	406.31
			TT	0.165	139	139.48
c.1571 G > A	G	0.893	GG	0.806	678	670.63
	A	0.107	AG	0.174	146	160.74
			AA	0.020	17	9.63
c.2776 A > G	A	0.819	AA	0.668	562	563.65
	G	0.181	AG	0.301	253	249.69
			GG	0.031	26	27.65

## Supplementary Materials

The following are available online at https://www.mdpi.com/2076-2615/10/4/671/s1, Table S1: The primers used for SNPs identification of bovine *FADS2* gene. Table S2: Effects of *FADS2* gene SNPs on milk production traits.
