# Supplementary material for: Polymorphisms in Fatty Acid Desaturase 2 Gene Are Associated with Milk Production Traits in Chinese Holstein Cows
Source: Animals (Basel). 2020 Apr 12;10(4):671. doi: 10.3390/ani10040671 (PMC7222784; doi:10.3390/ani10040671)
Supplement: Supplementary file 1 [file animals-10-00671-s001.pdf]

animals

Article

Supplementary Files: Polymorphisms in Fatty Acid Desaturase 2 Gene are Associated with Milk Production Traits in Chinese Holstein Cows

Mingxun Li [1,2], Qisong Gao [1], Mengqi Wang [1], Yan Liang [1], Yujia Sun [2], Zhi Chen [1,2], Huimin Zhang [1,2], Niel A. Karrow [3], Zhangping Yang [1,2] and Yongjiang Mao [1,2,*]

1 Key Laboratory of Animal Genetics & Breeding and Molecular Design of Jiangsu province, Yangzhou University, Yangzhou 225009, China; limingxun@live.com (M.L.); MZ120181011@yzu.edu.cn (Q.G.); mengqi.wang.1@ulaval.ca (M.W.); 15755081060@163.com (Y.L.); chenzhijerom@163.com (Z.C.); minmin-911@163.com (H.Z.); yzp@yzu.edu.cn (Z.Y.)

2 Joint International Research Laboratory of Agriculture and Agri-Product Safety of Ministry of Education of China, Yangzhou University, Yangzhou 225009, China; ysunshine30@outlook.com

3 Center for Genetic Improvement of Livestock, Department of Animal Biosciences, University of Guelph, Guelph, ON N1G 2W1, Canada; nkarrow@uoguelph.ca

* Correspondence: cattle@yzu.edu.cn

Received: 26 February 2020; Accepted: 10 April 2020; Published: 12 April 2020

Table S1. The primers used for SNPs identification of bovine *FADS2* gene.

Primers	Primer Sequences (5′-3′)	Annealing Temperature (°C)	Amplicon Size (bp)
P1	F: TGACTTGACTCCGAGCTTCA<br>R: CATCACTCAGCCTTCAGGAAC	60	1024
P2	F: CGCCTACCTCCACAAATGAA<br>R: ACCCAGGGAATCCAGCAC	59	471
P3	F: GGAGACAGAGGGAAAGAGTC<br>R: CAGTTCTCAGTGGGAGGG	56	317

Table S2. Effects of *FADS2* gene SNPs on milk production traits.

Snps	Genotypes	DHI Records Number	Test-Day Milk Yield (Kg) [1]	Fat Percentage (%) [1]	Protein Percentage (%) [1]	305-Day Milk Yield(Kg) [2]	305-Day Fat Yield(Kg) [2]	305-Day Protein Yield (Kg) [2]	Somatic Cell Score [1]
c.908 C>T (p.Ala294Val)	CC	6553	32.91 ^{Bb} ± 0.13	4.26 ^{Aa} ± 0.01	3.32 ± 0.00	9803.35 ^{Bb} ± 27.15	425.03 ^{Bb} ± 1.27	325.34 ^{Bb} ± 0.84	1.99 ^{Aa} ± 0.02
	CT	8687	33.41 ^{Aa} ± 0.11	4.21 ^{Bb} ± 0.01	3.32 ± 0.00	10013.89 ^{Aa} ± 23.37	428.52 ^{ab} ± 1.07	331.95 ^{Aa} ± 0.72	1.9 3^{ab} ± 0.01
	TT	3024	33.29 ^{ab} ± 0.19	4.26 ^{Aa} ± 0.02	3.33 ± 0.01	9959.33 ^{Aa} ± 40.77	430.64 ^{Aa} ± 1.88	331.16 ^{Aa} ± 1.27	1.88 ^{Bb} ± 0.02
	p		0.000	0.003	0.007	0.000	0.003	0.000	0.003
	α		0.13	0.01	0.01	53.34	2.68	2.22	−0.05
c.1571 G>A	GG	14578	33.25 ± 0.09	4.23 ± 0.01	3.32 ± 0.00	9949.14 ^{Aa} ± 18.26	427.71 ± 0.85	329.56 ± 0.57	1.94 ± 0.01
	GA	3313	33.1 ± 0.18	4.26 ± 0.01	3.34 ± 0.01	9886.85 ^{Bb} ± 38.07	428.08 ± 1.73	329.72 ± 1.15	1.99 ± 0.02
	AA	373	32.33 ± 0.51	4.31 ± 0.04	3.39 ± 0.02	9542.96 ^{Bb} ± 102.38	420.51 ± 4.85	322.99 ± 3.15	1.96 ± 0.07
	p		0.684	0.227	0.070	0.045	0.101	0.111	0.589
	α		−0.70	0.05	0.05	−313.76	−6.72	−5.99	−0.02
c.2776 A>G	AA	12110	33.12 ± 0.09	4.24 ± 0.01	3.33 ^{Aa} ± 0.00	9906.26 ± 20.01	426.05 ± 0.92	329.25 ± 0.62	1.94 ± 0.01
	AG	5619	33.37 ± 0.14	4.26 ± 0.01	3.32 ^{Aa} ± 0.00	9966.57 ± 29.31	431.37 ± 1.35	330.23 ± 0.91	1.96 ± 0.02
	GG	518	33.43 ± 0.44	4.13 ± 0.03	3.23 ^{Bb} ± 0.01	10063.15 ± 95.06	425.49 ± 4.95	326.47 ± 3.22	1.94 ± 0.05
	p		0.142	0.051	0.000	0.056	0.200	0.142	0.303
	α		0.09	−0.10	−0.08	90.02	−3.85	−2.90	−0.01

[1] Test-day records; [2] Data for the entire 305-d lactation; α= allele substitution effects; Means with different uppercase superscripts within a column differ ($p < 0.01$); Means with different lowercase superscripts within a column differ ($p < 0.05$); p-value shows the significance for SNPs effects on production traits.

© 2020 by the authors. Licensee MDPI, Basel, Switzerland. This article is an open access article distributed under the terms and conditions of the Creative Commons Attribution (CC BY) license (http://creativecommons.org/licenses/by/4.0/).
